# SOCIAL ENGAGEMENT, COGNITIVE IMPAIRMENT, AND MOBILITY IN THE BOSTON REHABILITATIVE IMPAIRMENT STUDY OF THE ELDERLY

**DOI:** 10.1093/geroni/igz038.1607

**Published:** 2019-11-08

**Authors:** Lien Quach, David Gagnon, Jennifer Moye, Kelly Cho, Jonathan Bean

**Affiliations:** 1 Veterans Administration, Boston, Massachusetts, United States; 2 VA Boston Healthcare System, Boston, Massachusetts, United States

## Abstract

This study examined how social engagement (SE) and mild cognitive impairment (MCI) influence changes in mobility over three years of follow-up. We performed a secondary analysis of longitudinal data among primary care patients aged >64 years (N=430). Mobility outcomes include performance-based function via the Short Physical Performance Battery (SPPB) and patient reported function via the Late-Life Function Instrument (LLFI). Independent variables include: 1) MCI determined by a comprehensive cognitive battery and scores 1.5 SD < age-adjusted mean; 2) SE measured by standardized self-report of social activities. Multivariate linear mixed regression models demonstrate that MCI is associated with lower scores on SPPB and LLFI (β= -0.76, p<0.0001; β= -1.47, p=0.005 respectively). SE is associated with higher scores on SPPB and LLFI (β=0.56, p<0.0001; β= 1.95, p<0.0001 respectively), and partially mediates the association between MCI and on each outcome. SE is linked to mobility decline especially among participants with MCI.

